# Availability and the quality of key newborn data within routine health facility data: findings of the IMPULSE observational study in the Central African Republic, Ethiopia, Tanzania, and Uganda

**DOI:** 10.7189/jogh.15.04359

**Published:** 2025-12-05

**Authors:** Rornald Muhumuza Kananura, Dalena Paolo, Lorenzo Giovanni Cora, Firehiwot Abathun, Ousman Mouhamadou, Jacqueline Minja, Mary Ayele, Francesca Tognon, Ilaria Mariani, Sara Geremia, Giovanni Putoto, Donat Shamba, Louise Tina Day, Peter Lochoro, Richard Mugahe, Chris Ebong, Marzia Lazzerini, Peter Waiswa

**Affiliations:** 1 Demographic Dynamic and Population Health Unit, African Population and Health Research Center, Dakar, Senegal; 2 Makerere University Centre of Excellence for Maternal Newborn & Child Health, Kampala, Uganda; 3 Institute for Maternal and Child Health IRCCS Burlo, Garofolo, Trieste, Italy; 4 University of Trieste, Trieste, Italy; 5 WHO Collaborating Centre for Maternal and Child Health, Institute for Maternal and Child Health IRCCS Burlo, Garofolo, Trieste, Italy; 6 Doctors with Africa CUAMM, Addis Ababa, Ethiopia; 7 Doctors with Africa CUAMM, Bangui, Centra African Republic; 8 Ifakara Health Institute, Dares Salaam, Tanzania; 9 Doctors with Africa CUAMM, Padua, Italy; 10 London School of Hygiene & Tropical Medicine, London, UK; 11 Doctors with Africa CUAMM, Kampala, Uganda; 12 Ministry of Health, Kampala, Uganda

## Abstract

**Background:**

With declining funding for population-based household surveys, routine health facility data offer a promising alternative for tracking newborn health and service quality. However, their utility depends on data quality. We assessed the quality of ten data elements within routine health information systems in the Central African Republic (CAR), Ethiopia, Tanzania, and Uganda, seven of which align with the Every Newborn Action Plan core newborn indicators.

**Methods:**

We conducted a cross-sectional study in 97 emergency obstetric and newborn care facilities across 4 countries between November 2022 and July 2024. We extracted three months of routine register and summary report data on ten maternal and newborn elements (two denominators, three outcome numerators, five newborn care interventions) and one tracer maternal indicator. We evaluated data quality on four dimensions (availability, completeness, accuracy, and internal consistency) and measured internal consistency using the ratio of (total births − live births)/stillbirths, with a value of 1 suggesting ideal internal consistency.

**Results:**

Denominator completeness exceeded 90% in Uganda and Tanzania, but was lower in the CAR (87%) and Ethiopia (82%). Impact numerator completeness averaged 79% for neonatal mortality and 81% for low birth weight, with Ethiopia performing worst, with scores of 45% and 32%, respectively). Completeness for newborn interventions (early breastfeeding, kangaroo mother care, bag-mask ventilation, sepsis management) remained below 90%, with the CAR lacking neonatal sepsis data and Ethiopia lacking early breastfeeding data. Accuracy was poor: concordance between register recounts and summary reports ranged from 9% to 40%. Internal consistency checks revealed mismatches in 80% of facilities, including negative ratios in Uganda and ratios >1 in the CAR.

**Conclusions:**

Significant gaps in completeness, accuracy, and internal consistency undermine the reliability of newborn and stillbirth data in routine health information systems, highlighting a need for their strengthening, the integration of standardised newborn indicators, and institutionalized quality verification processes to ensure timely, reliable, and actionable data for improving newborn care.

In recent decades, there has been a growing recognition of the importance of quality newborn healthcare at the facility level [1]. Despite efforts in low- and middle-income countries (LMIC), studies have revealed persistent gaps and inequities in newborn quality of care [2–4]. In acknowledgement of this issue, a consensus emerged on the need for enhanced data collection, with a focus on service coverage, quality, and outcomes to drive accountability and action [5–7]. Research has shown that timely, high-quality maternal and newborn data at all hierarchical levels are critical for enhancing care quality and strengthening health systems [5,6,8–10]. Yet, paradoxically, countries with the weakest data on service coverage and quality bear the highest burden of global newborn mortality and stillbirths [5,8,11–14].

A need to improve the quality of routine health information systems (RHIS) for maternal and newborn data has been highlighted by the Every Woman, Every Newborn, Everywhere Plan (previously known as the Every Newborn Action Plan (ENAP) [10,15–17], World Health Organization (WHO), and the United Nations Children’s Fund, emphasising the significance of high-quality, timely, and reliable data for improving healthcare outcomes, which is critical for monitoring progress towards Sustainable Development Goals (SDGs). Improving the measurement of indicators and the quality of data has been well studied as a key driver for HIV, immunisation programmes, and COVID-19 programming [18–20]. Nonetheless, substantial gaps in health facility documentation and reporting persist in LMICs [5,21–26], despite the RHIS’ potential benefits for monitoring service quality and promoting accountability, and the regular availability of its data for timely action by health workers, facility/district managers, and policymakers [15,27–29].

Moreover, the expansion of digital platforms, such as the District Health Information Software 2 (DHIS2) across sub-Saharan Africa enhances the potential of harmonised health management information system (HMIS) data elements to improve data availability at subnational and regional level estimates. Specifically, the DHIS2 is now being implemented in 130 countries in Africa and Asia as their routine data management system [30]. With standardised newborn health indicators, the RHIS can track progress within and across countries, complementing household surveys such as the Demographic and Health Surveys and Multiple Indicator Cluster Surveys. While these surveys have long provided key health statistics in LMICs, they occur only every five years, often lack subnational detail, and omit many indicators on care quality and intervention coverage [31]. These challenges are further compounded by declining global funding for large-scale surveys, raising concerns about their long-term sustainability.

Although countries have established core indicators and targets, the utility of RHIS data is contingent upon their completeness, availability, accuracy, and internal consistency [32] that remain weak in many settings and require targeted improvement efforts [33]. Research such as the EN-BIRTH study has identified challenges in information systems, including difficulties in calculating indicators, inadequacies in data flow and resources, weaknesses in standardized case definitions, and leadership issues [5]. Poor-quality RHIS data directly undermine how health systems function in practice. When key events such as live births or neonatal deaths go undocumented, health workers and managers lose the ability to monitor coverage, identify service gaps, and may inadvertently mask deeper shortfalls in service availability. Likewise, underreporting or overreporting skews resource allocation, while internal inconsistencies expose potential lapses in care quality.

The body of evidence presented above underscores the need to assess and strengthen the quality of routine newborn and stillbirth data collected through HMIS platforms. In response to these challenges, the Improving Quality and Use of newborn indicators (IMPULSE) phase 1 study was designed to generate evidence on the quality and use of RHIS for newborn and stillbirths in health facilities and data offices. Here we present the findings findings of an assessment of the quality of routine health facility data in four sub-Saharan African countries: the Central African Republic (CAR), Ethiopia, Tanzania, and Uganda. The assessment comprised 10 key newborn health indicators, seven of which were adapted from the ENAP framework [31], and focussed on four key dimensions: availability of the indicators in the aggregated official reports, completeness (facility source registers), accuracy (concordance between source records and reported summaries), and internal consistency (coherence among related birth outcome data elements).

## METHODS

### Study design

We conducted this cross-sectional study in four sub-Saharan African countries: the CAR, Ethiopia, Tanzania, and Uganda. Its design was guided by the Performance of Routine Information System Management (PRISM) conceptual framework [34], selected for its comprehensive and structured approach to assessing the performance of RHIS, including technical, organisational, and behavioural determinants of data quality and use. This makes it particularly suited for evaluating complex health information systems in LMICs, where challenges are multifactorial.

We selected 12 regions and 4 city administrations as study sites based on three criteria:

1. heterogeneity in performance and context, including the selection of underperforming regions for maternal and neonatal mortality and/or those located in humanitarian or hard-to-reach settings;

2. logistical feasibility, particularly for regions with an existing presence of the implementing agency (Doctors with Africa *Collegio Universitario Aspiranti Medici Missionari* (*CUAMM*)) or those accessible by road;

3. selection of region based on prioritisation by the respective ministries of health.

We chose a minimum of 19 health facilities in each country using the PRISM-adapted lot quality assurance sampling methodology, which enables identification of performance gaps across multiple facilities without requiring a full census of all health facilities in a region [35]. In total, 97 public and private-not-for-profit health facilities were assessed: 93 providing comprehensive emergency obstetric and newborn care services across the 4 study countries and 4 high-volume basic emergency obstetric and newborn care facilities in the CAR (Table S1 in the **Online Supplementary Document**), which were included due to contextual constraints in service availability. We excluded facilities located in conflict-affected areas, those inaccessible due to poor road infrastructure, or those that declined participation.

### Data collection

The study coordinator in each country trained three to six data collectors and supervised practice sessions at non-study sites before actual data collection commenced. Data were collected between November 2022 and July 2024 by teams of two to three trained researchers, who visited each site over a period of one to three days. Data were collected from routine registers and summary reports using password-protected tablets equipped with SurveyCTO forms (Dobility, Inc., Washington, D.C., USA) and subsequently uploaded to a secure, encrypted server. For this study, data were collected predominantly using Every Newborn-Measurement Improvement for Newborn & Stillbirth Indicators (EN-MINI) Tool 2B, ‘Performance Diagnostic for Routine Health Information Systems, version 2’ [35].

### Data analysis

We present our analysis based on data collected to assess the quality of ten maternal and newborn health data elements: two denominators (total births and live births); three impact numerators (stillbirths, institutional neonatal deaths, and low birth weight (LBW)); and five newborn health practices and interventions (early initiation of breastfeeding, bag-mask ventilation, initiation of kangaroo mother care (KMC), and management of neonatal sepsis). We also included one tracer maternal data element (administration of uterotonics to prevent postpartum haemorrhage) because it is closely linked to quality intrapartum care and serves as a maternal health intervention with direct implications for newborn survival. These data elements allow for the calculation of eight key indicators, prioritised under the ENAP strategy.

We assessed the availability and quality of routine newborn health data over a three-month reference period by reviewing both source registers and facility reporting tools (**Table 1**). The data quality assessment focused on four core dimensions: availability, completeness, accuracy, and internal consistency. For internal consistency, we calculated the ratio of (total births − live births)/stillbirths, which, under correct reporting, should equal 1. A value of 1 indicates perfect alignment between the reported counts of total births, live births, and stillbirths, with deviations from 1 suggesting internal inconsistency. A ratio >1 indicates that the difference between total and live births exceeds reported stillbirths, suggesting underreporting or misclassification of stillbirths. A ratio <1 indicates that the reported stillbirths exceed the difference between total and live births. A negative ratio – where live births exceed total births – is logically impossible and reflects severe reporting errors, likely due to misclassification, aggregation mistakes, or inconsistent case definitions.

**Table 1:** Descriptions of data quality dimensions and analysis approaches

Dimensions	Description
Completeness of source register	A register was deemed complete if no missing values were observed for each of the 10 data elements.
	Numerator: number of facilities with complete entries for each of the indicators in the source registers.
	Denominator: total number of health facilities surveyed.
Availability of reported data	Availability was defined as the reporting of each of the ten data elements in the facility’s monthly reporting aggregated summary forms.
	Numerator: number of facilities that reported each of the ten data elements.
	Denominator: total number of health facilities surveyed.
Accuracy of reported data	Assessed through a recount of individual records on each indicator in the facility registers, which was then compared with the corresponding aggregated figures reported in monthly summary forms.
	Numerator: number of facilities without any discrepancy between source register data and monthly reports for each of the 10 indicators.
	Denominator: total number of health facilities surveyed.
Internal consistency	Assessed using aggregated monthly reports submitted by health facilities to the district and focused on birth outcome indicators: total births, live births, and stillbirths. According to standard definitions, total births should equal the sum of live births and stillbirths, and therefore, the ratio of (total births − live births)/stillbirths should be one.
**Indicators**	
Denominator	Total births and live births
Impact numerators	Stillbirths, institutional neonatal deaths, low birth weight
Intervention numerators	Early initiation of breastfeeding, bag-mask-ventilation, kangaroo mother care initiation, neonatal sepsis, and uterotonics to prevent postpartum haemmorhage

## RESULTS

### Characteristics of the sample

At least 70% of the 97 health facilities in the CAR, Ethiopia, and Tanzania were in urban settings, while 57% of the facilities in Uganda were situated in rural areas. Across all four countries, facilities were predominantly government or public health facilities (Table S1 in the **Online Supplementary Document**).

### Availability and quality of newborn denominator and impact numerator data elements at health facility level

**Figure 1:**
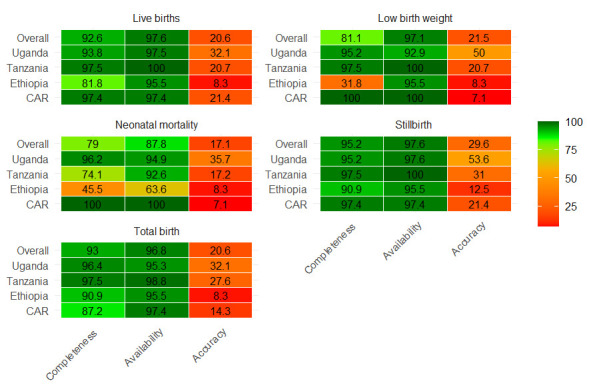
Availability and quality of newborn denominator and impact numerator data elements at health facility level.

The completeness of total birth and livebirth denominator data elements was at least 90%, except in the CAR, where completeness of total birth was 87%, and in Ethiopia, where completeness of live births was 82% (**Figure 1**). The overall completeness of impact numerators – neonatal mortality and LBW – was suboptimal, at 79% and 81%, respectively. Completeness was particularly low in Ethiopia, where neonatal mortality and LBW were recorded at 45% and 32%, respectively. Similarly, completeness for neonatal mortality in Tanzania was suboptimal, at 74%.

In all countries, the accuracy of newborn health outcomes data was a major challenge. Perfect concordance between register recounts and monthly aggregated reports ranged from just 9% to 40% (**Table 2**). Allowing a ±10% tolerance, LBW reporting accuracy improved – from 21% in Ethiopia to 67% in Uganda – yet total births and live births remained below 90% accuracy across all settings. Stillbirth reporting accuracy ranged from 21% in the CAR to 53% in Uganda, while neonatal mortality accuracy varied from 7% (CAR) to 36% (Uganda).

**Table 2:** Accuracy of newborn health outcomes’ data

	Perfect match	5% variance	10% variance
**Total births**			
Overall	22.1	69.5	74.7
CAR	14.3	71.4	71.4
Ethiopia	8.3	54.2	54.2
Tanzania	27.6	75.9	89.7
Uganda	32.1	75.0	78.6
**Live births**			
Overall	21.1	72.6	76.8
CAR	21.4	71.4	78.6
Ethiopia	8.3	54.2	54.2
Tanzania	20.7	82.8	86.2
Uganda	32.1	78.6	85.7
**Stillbirth**			
Overall	31.6	32.6	36.8
CAR	21.4	21.4	21.4
Ethiopia	12.5	16.7	29.2
Tanzania	31.0	31.0	34.5
Uganda	53.6	53.6	53.6
**Low birth weight**			
Overall	24.2	31.6	38.9
CAR	7.1	14.3	35.7
Ethiopia	8.3	12.5	20.8
Tanzania	20.7	24.1	27.6
Uganda	50.0	64.3	67.9
**Institutional neonatal deaths**			
Overall	18.9	20.0	21.1
CAR	7.1	7.1	7.1
Ethiopia	8.3	12.5	12.5
Tanzania	17.2	17.2	17.2
Uganda	35.7	35.7	35.7

### Internal inconsistencies in the related denominator and impact data elements

Overall, 80% of the health facilities demonstrated internal inconsistencies in the reported numbers of stillbirths, total births, and live births during the three months preceding data collection. In each country, at least 70% of the facilities exhibited such discrepancies, with Ethiopia showing the highest proportion (Figure S1 in the **Online Supplementary Document**).

**Figure 2:**
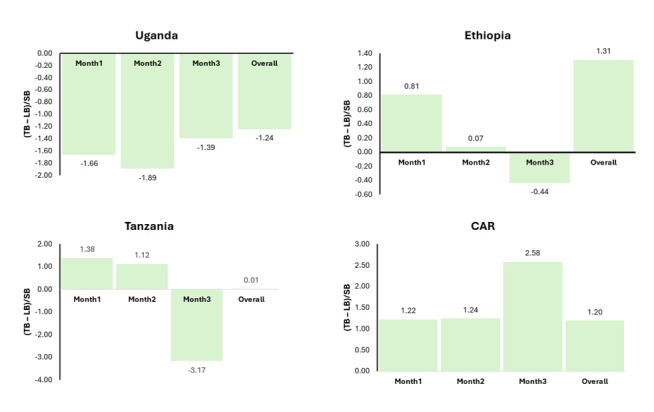
Inconsistence in the reporting of TB, LB, and SB. Ratios were calculated as (TB-LB)/SB. Ratios of 1, >1, <1 indicate consistent reporting, possible underreporting of stillbirths/documentation gaps, or overreporting of stillbirths or denominator errors, respectively. A negative ratio indicates invalid reporting: LB>TB. A tolerance of ±10 is used as the threshold for identifying internal inconsistencies. LB – live birth, SB – stillbirth, TB – total birth.

In Uganda, the ratio of the difference between total births and live births to stillbirths remained consistently negative – by at least 39% – over the three months preceding data collection, indicating that the number of live births recorded in the study health facilities exceeded the total number of reported births (**Figure 2**). Conversely, the difference between total births and live births in the CAR exceeded the number of stillbirths by at least 20% throughout the same period. In Tanzania, this ratio was > 1 during the first two months, indicating that the difference between total births and live births exceeded the number of stillbirths. By the third month, however, the ratio fell below 1, suggesting that stillbirths outnumbered the difference between total births and live births.

### Availability and the quality of data elements for essential newborn health practices at facility level

**Figure 3:**
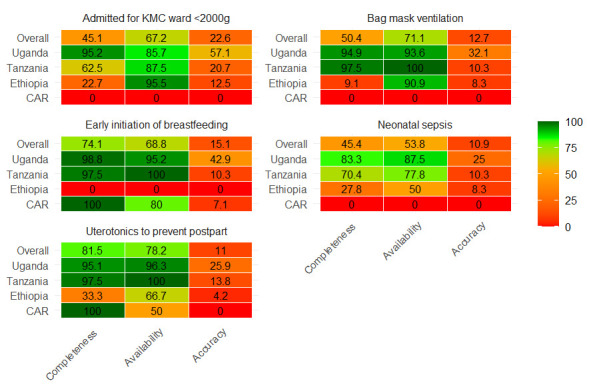
Quality of data for essential newborn health practice at facility level.

The availability and completeness of all the newborn health practices indicators was less than 90% (**Figure 3**). The overall completeness for numerators: neonatal sepsis, initiation of KMC, and bag mask ventilation were substantially lower, at 45%, 45%, and 50% respectively. Moreover, documentation and reporting on bag mask ventilation, KMC admissions, and neonatal sepsis were entirely absent in the CAR, while documentation and reporting on early initiation of breastfeeding were not available in Ethiopia.

The accuracy of reported newborn health intervention data elements was suboptimal. Exact agreement between register recounts and aggregated RHIS figures fell below 20% for every indicator (**Table 3**). Allowing a ±10% tolerance, only 55% of Tanzanian and 78% of Ugandan facilities met the cutoff for early breastfeeding accuracy, compared with just 21% in the CAR. Uterotonic administration accuracy was under 10% in all countries except Uganda (81%). Accuracy for neonatal sepsis management ranged from 12% in Ethiopia to 29% in Uganda, while bag-mask ventilation reporting accuracy ranged from 8% in Ethiopia to 32% in Uganda.

**Table 3:** Accuracy of newborn health interventions’ data

	Perfect match	5% variance	10% variance
**Early breastfeeding**			
Overall	16.8	37.9	43.2
CAR	7.1	14.3	21.4
Ethiopia	NA*	NA*	NA*
Tanzania	10.3	48.3	55.2
Uganda	42.9	71.4	78.6
**Bag mask ventilation**			
Overall	14.7	15.8	23.2
CAR	NA*	NA*	NA*
Ethiopia	8.3	8.3	8.3
Tanzania	10.3	13.8	27.6
Uganda	32.1	32.1	32.1
**KMC initiation**			
Overall	26.3	29.5	32.6
CAR	NA*	NA*	NA*
Ethiopia	12.5	12.5	12.5
Tanzania	20.7	24.1	24.1
Uganda	57.1	64.3	71.4
**Neonatal Sepsis**			
Overall	12.6	14.7	15.8
CAR	NA*	NA*	NA*
Ethiopia	8.3	12.5	12.5
Tanzania	10.3	10.3	13.3
Uganda	25	28.6	28.6
**Uterotonic to prevent postpartum haemorrhage**			
Overall	12.6	37.9	41.1
CAR	0.0	7.1	7.1
Ethiopia	4.2	4.2	4.2
Tanzania	13.8	4.2	4.2
Uganda	25.9	77.8	81.5

NA – not applicable

*Data element not documented/available.

## DISCUSSION

Our assessment of key newborn health data elements within RHIS across the study countries provides insights into the state of newborn data quality in sub-Saharan Africa. To our knowledge, this is the first and largest multi-country assessment examining the quality of newborn and stillbirth data recorded in health facility registers and reporting forms in the African region. Our findings reveal persistent challenges in data quality, with notable variation across countries and across the different dimensions assessed.

### Availability and quality of newborn denominator and impact numerator data elements at health facility level

There was notable heterogeneity in the completeness of denominator data elements, such as live births and total births. For instance, Ethiopia demonstrated poor completeness of live births (81%), while the CAR showed suboptimal completeness of total births (87%). Moreover, completeness of neonatal mortality and LBW data varied widely, with Ethiopia and Tanzania reporting particularly low completeness for newborn mortality (45% and 74%, respectively), and Ethiopia again performing poorly for LBW documentation. Across all four countries, fewer than half of facilities achieved accurate reporting for newborn outcomes and care practices, echoing concerns from earlier reviews and country-level studies [5,7,36]. Internal consistency checks reinforced these weaknesses: while the ideal ratio would be (total births − live births)/stillbirths = 1, approximately 80% of facilities deviated from this benchmark, with negative ratios observed in Uganda and ratios >1 in the CAR.

The identified discrepancies in recording both numerator and denominator elements for birth outcomes critically undermine the validity of neonatal mortality, stillbirth, and LBW prevalence estimates. Live and total births form the denominators of most newborn coverage indicators, so their underreporting or misclassification systematically biases national and global statistics. Likewise, inaccurate numerator reporting, for example, of stillbirths or LBW, lead to misleading coverage rates. These inconsistencies often reflect a combination of factors.

Errors frequently arise during the transfer of data from facility registers to departmental and facility summary forms, where omissions, transcription mistakes, and misinterpretations occur. Limited knowledge among health workers about standardised indicator definitions, for instance, the cutoff for LBW or the clinical criteria for stillbirth, further contributes to inconsistent documentation [37]. Additional discrepancies are introduced during aggregation into monthly reporting forms for DHIS2 entry, through manual calculations, incomplete transfer, or double counting. Beyond unintentional mistakes, deliberate misclassification adds another layer of inaccuracy. Health workers may intentionally reclassify outcomes – for instance, reporting stillbirths as neonatal deaths – to avoid blame, minimise negative performance indicators, or navigate sociocultural stigma [25,37]. Such errors, rarely corrected due to weak feedback mechanisms, become institutionalised in national statistics, undermining the reliability of RHIS and distorting estimates of newborn outcomes. In Uganda, inconsistencies of reported live births exceeding the total births likely reflect misclassification of stillbirths as neonatal deaths, transcription errors, or inconsistent use of definitions across registers and reports. By contrast, the month-to-month fluctuations observed in Ethiopia and Tanzania point to aggregation errors and instability in reporting practices.

In fragile health system contexts such as the CAR, where the gap between total and live births exceeded recorded stillbirths by more than 20%, systemic underreporting of stillbirths or breakdowns in documentation are compounded by structural weaknesses in health information systems. In fragile states, these challenges are amplified by shortages of trained staff, high turnover, weak supervision, and the absence of standardised registers or digital tools. In such settings, facilities often rely on improvised records, increasing the risk of omissions and errors, while conflict and humanitarian crises further disrupt data tools, staff capacity, and accountability mechanisms. As a result, documentation problems are not only technical, but systemic, raising serious concerns that stillbirths and other newborn outcomes are substantially underestimated in the CAR and similar contexts.

### Availability and the quality of data elements for essential newborn and maternal health practices at facility level

The completeness of newborn intervention data within national RHIS platforms was strikingly weak. This is consistent with findings from the EN-BIRTH study, which showed that service delivery and intervention coverage indicators are less reliably documented than outcome or denominator data [5]. These gaps do not necessarily reflect the absence of services; rather, they often stem from poor documentation practices, weak reporting systems, or misaligned tools. For example, the absence of early-breastfeeding entries in Ethiopia is unlikely to indicate non-provision, given the country’s long-standing breastfeeding promotion efforts, but instead points to recordkeeping deficiencies. Similarly, the lack of data on KMC, neonatal sepsis management, and bag-mask ventilation in the CAR could reflect true service gaps, systemic documentation failures, or both. Distinguishing among these possibilities is critical for programmatic response: while service gaps require investment in preparedness and essential equipment, weak documentation calls for improved tools, training, and accountability mechanisms.

Accuracy challenges reinforce this interpretation. Across all intervention data elements, exact agreement between register recounts and RHIS reports was rare, with most countries falling well below acceptable thresholds. These inconsistencies highlight not only systemic weaknesses in data management, but also the pressures faced by frontline health workers, who often balance heavy clinical workloads with documentation duties. As seen in other studies, data completeness and accuracy depend heavily on the availability of functional equipment, clearly defined service protocols, and supportive supervision [36]. Addressing these weaknesses therefore requires strategies that go beyond data audits to include investments in service readiness, provision of user-friendly documentation tools, and creating an enabling environment where accurate reporting is valued and rewarded.

Collectively, these weaknesses compromise not only the accuracy of national and global estimates, but also the ability of health systems to track progress, allocate resources effectively, and ensure accountability.

### Implications

While RHIS data enables real-time monitoring of newborn health services, the quality concerns identified in this study cast doubt on their reliability for decision-making. Inadequate documentation and reporting of newborn interventions in global and national frameworks hinder targeted care for vulnerable newborns. Improving outcomes requires strengthening both data systems and health services throughout the system to ensure equitable, high-quality newborn care.

For ministries of health and national policymakers, our findings highlight the urgent need to strengthen the integration of newborn indicators into RHIS platforms, while recognising that priorities will vary across settings depending on which dimensions of data quality are most problematic. In the CAR, the complete absence of data on KMC, neonatal sepsis, and bag-mask ventilation points to the need for both service preparedness and standardised documentation procedures. Integrating ENAP core indicators into registers must coincide with ensuring facilities are equipped to deliver these services. In Ethiopia, where completeness of neonatal mortality and LBW data was especially low, efforts should focus on clarifying case definitions, reinforcing documentation of birth weights, and embedding newborn indicators into existing maternal health reporting tools. In Tanzania and Uganda, where availability and completeness were relatively strong, the main challenge remains poor accuracy. Here, ministries should prioritise systematic audits comparing aggregated reports against registers, institutionalise EN-MINI-type data quality checks, and strengthen district-level capacity to act on discrepancies. Across all four countries, building capacity for data analysis at the district level is critical for evidence-based planning, equitable resource allocation, and monitoring of service quality.

For health workers and facility managers, data quality begins at the point of care. In Uganda and Tanzania, where reporting is relatively complete, interventions should focus on strengthening staff capacity to improve accuracy, by maintaining thorough and precise records, minimising aggregation errors, and ensuring consistent transfer of data from registers to summary forms. In the CAR, where data gaps may partly reflect lack of service delivery, frontline staff need supportive supervision and standardised recording formats that encourage routine documentation, even in resource-constrained settings. In Ethiopia, sustained training on the definitions of stillbirth and neonatal mortality is necessary to minimise misclassification and ensure consistency across facilities. Building a culture of accountability in all countries, where staff recognise the link between reliable data and improved newborn outcomes, will require continuous engagement through supportive supervision, mentorship, and recognition. Regular participation in facility- and district-level data review meetings can provide opportunities to identify errors, address inconsistencies, and link data use to quality improvement efforts.

For donors and development partners, these findings underscore the importance of sustained investments in RHIS strengthening. With global funding for large-scale household surveys becoming increasingly uncertain, development partners should prioritize facility-based RHIS as a complementary and sustainable source of data. Support should also focus on digital innovations, such as automated validation checks within DHIS2 and integration of EN-MINI tools, which can provide real-time insights on completeness and internal consistency. Equally important is investment in capacity-building initiatives that empower facility and district staff to generate, review, and apply data effectively in decision-making processes.

### Strengths and limitations

This study’s strength lies in its assessment of both primary data sources and aggregated reports across basic or comprehensive emergency obstetric and newborn care facilities in four countries, providing a nuanced understanding of data quality at multiple levels of the health system. A further strength is the use of the standardised EN-MINI Tool 2B for data collection [7], which enhances comparability and supports the generalizability of findings across sub-Saharan Africa.

A key limitation, however, is the focus on *CUAMM*-supported regions, which are often located in humanitarian or low-resource settings where health systems experience chronic constraints. While this may limit representativeness, it is important to recognise that *CUAMM*-supported facilities are also likely to be better resourced than many other facilities within these countries. The persistence of significant data quality weaknesses in such facilities, therefore, suggests that the broader national situation may be even more concerning. Conversely, a focus on humanitarian and resource-constrained settings might also overstate gaps in comparison to more stable regions. We therefore interpret the findings as indicative rather than nationally representative, while noting that documentation gaps and reporting inconsistencies are widely recognised across LMIC facility-based RHIS. Therefore, while we recommend extending the assessment to additional facilities, it is reasonable to expect that similar or greater challenges would be observed across health facilities in LMICs more generally.

## CONCLUSIONS

Our findings indicate that inaccuracies, incompleteness, and internal inconsistencies within facility-level data compromise the validity of statistics reported through national health information systems (DHIS2). Nonetheless, with the widespread uptake of facility-based deliveries and antenatal care across sub-Saharan Africa, high-quality RHIS data offer opportunities for timely and actionable insights. This is especially relevant and timely in the current context, where LMICs are increasingly rely on RHIS in the face of declining global funding for large-scale household surveys such as DHS. Achieving the ENAP accountability agenda hinges on fully integrating newborn health indicators into routine reporting and embedding systematic quality checks. Health ministries and implementing partners should institutionalize regular RHIS audits, standardise and harmonise ENAP measures within facility registers and DHIS2, and invest in the capacity of frontline staff to capture, analyse, and act on data.

## Additional material


Online Supplementary Document
