# Supplementary material for: Availability and the quality of key newborn data within routine health facility data: findings of the IMPULSE observational study in the Central African Republic, Ethiopia, Tanzania, and Uganda
Source: J Glob Health. 2025 Dec 5;15:04359. doi: 10.7189/jogh.15.04359 (PMC12677238; doi:10.7189/jogh.15.04359)

Table S1: Characteristics of the study health facilities

	CAR	Ethiopia	Tanzania	Uganda
**Characteristic**	***n (%)***	***n (%)***	***n (%)***	***n (%)***
Total health facility	***14***	***24***	***29***	***30***
IMPULSE classification				
Third level of referral hospital	4 (28.6)	3 (12.5)	5 (17.2)	5 (16.7)
Second level of referral hospital	3 (21.4)	10 (41.7)	15 (51.7)	12 (40)
First level of referral hospital	7 (50)	11 (45.8)	9 (31.1)	13 (43.3)
PRISM classification				
National referral hospital	3 (21.4)	1 (4.2)	2 (6.9)	1 (3.4)
Regional referral hospital	1 (7.2)	2 (8.3)	3 (10.3)	4 (13.3)
District hospital	3 (21.4)	10 (41.7)	15 (51.7)	12 (40)
Health centre	7 (50)	11 (45.8)	9 (31.1)	13 (43.3)
Health clinic	0 (0.0)	0 (0.0)	0 (0.0)	0 (0.0)
**Urban/rural**				
Urban	13 (92.9)	18 (75)	21 (72.4)	13 (43.3)
Rural	1 (7.1)	6 (25)	8 (27.6)	17 (56.7)
**Managing authority**				
Government/public	12 (85.8)	18 (75)	22 (75.8)	23 (76.7)
Private	1 (7.1)	5 (20.8)	3 (10.3)	0 (0.0)
Not-for-profit	1 (7.1)	1 (4.2)	4 (13.9)	7 (23.3)
**Region**				
Bangui City	3 (21.4)	-	-	-
Health region 1	3 (21.4)	-	-	-
Health region 2	4 (28.6)	-	-	-
Health region 7	4 (28.6)	-	-	-
Addis Ababa City Administration	-	3 (12.5)	-	-
Oromia	-	9 (37.5)	-	-
Amhara and Gambella	-	3 (12.5)	-	-
South Ethiopia and Sidama	-	9 (37.5)	-	-
Dar es Salaam City	-	-	2 (6.9)	-
Iringa	-	-	10 (34.5)	-
Shinyanga	-	-	9 (31)	-
Simiyu	-	-	8 (27.6)	-
Lango	-	-	-	10 (33.3)
Karamoja	-	-	-	8 (26.7)
West-Nile	-	-	-	11 (36.7)
Kampala City	-	-	-	1 (3.3)

Figure S1: Percentage of health facilities with internal inconsistency in the reporting of stillbirth, total birth, and live birth within the three months preceding data collection

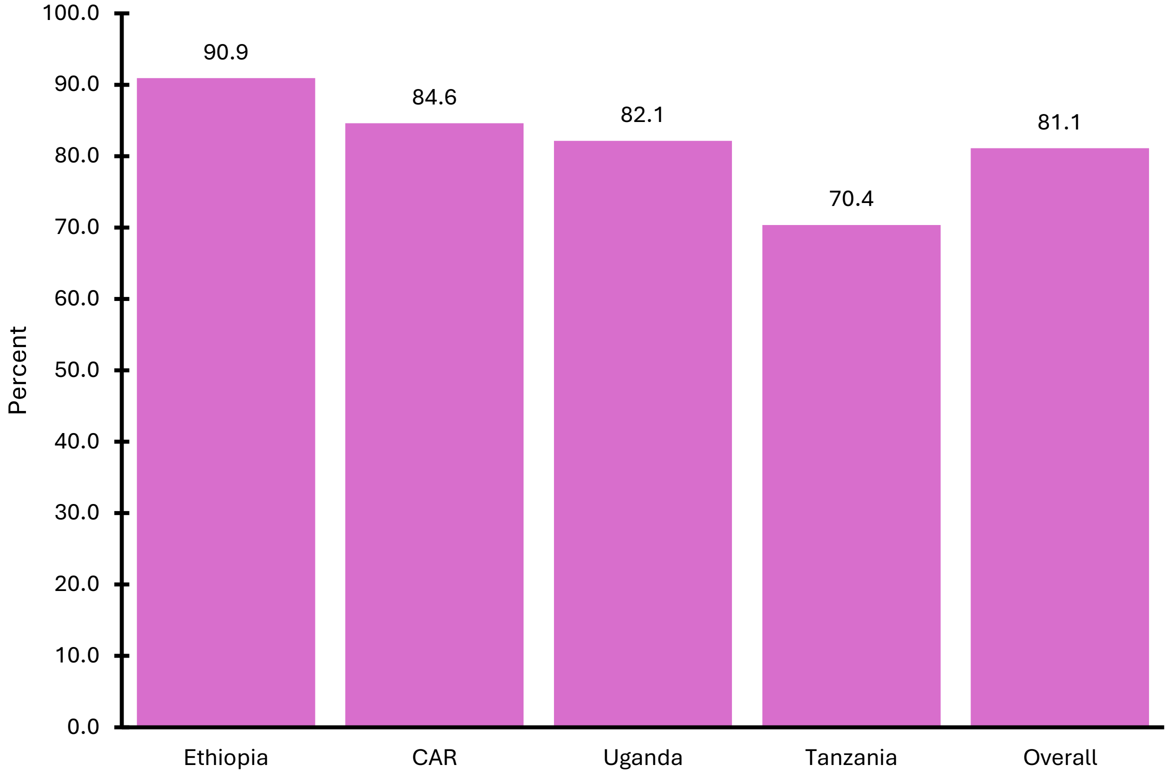

Supplement: Online Supplementary Document [file jogh-15-04359-s001.pdf]
